# Maternal overweight is not an independent risk factor for increased birth weight, leptin and insulin in newborns of gestational diabetic women: observations from the prospective *‘EaCH’* cohort study

**DOI:** 10.1186/s12884-018-1889-8

**Published:** 2018-06-20

**Authors:** Raffael Ott, Jens H. Stupin, Andrea Loui, Elisabeth Eilers, Kerstin Melchior, Rebecca C. Rancourt, Karen Schellong, Thomas Ziska, Joachim W. Dudenhausen, Wolfgang Henrich, Andreas Plagemann

**Affiliations:** 1 Clinic of Obstetrics, Charité – Universitätsmedizin Berlin, corporate member of Freie Universität Berlin, Humboldt-Universität zu Berlin, and Berlin Institute of Health, Campus Virchow-Klinikum, Berlin, Germany; 2 Division of ‘Experimental Obstetrics, Charité – Universitätsmedizin Berlin, corporate member of Freie Universität Berlin, Humboldt-Universität zu Berlin, and Berlin Institute of Health, Campus Virchow-Klinikum, Berlin, Germany; 3 Department of Neonatology, Charité – Universitätsmedizin Berlin, corporate member of Freie Universität Berlin, Humboldt-Universität zu Berlin, and Berlin Institute of Health, Campus Virchow-Klinikum, Berlin, Germany

**Keywords:** Gestational diabetes mellitus, Maternal overweight/obesity, Gestational weight gain, Maternal glucose, Birth weight, Cord-blood insulin, Cord-blood leptin, Newborn outcomes, Perinatal programming

## Abstract

**Background:**

Both gestational diabetes mellitus (GDM) as well as overweight/obesity during pregnancy are risk factors for detrimental anthropometric and hormonal neonatal outcomes, identified to ‘program’ adverse health predispositions later on. While overweight/obesity are major determinants of GDM, independent effects on critical birth outcomes remain unclear. Thus, the aim of the present study was to evaluate, in women with GDM, the relative/independent impact of overweight/obesity vs. altered glucose metabolism on newborn parameters.

**Methods:**

The prospective observational ‘Early CHARITÉ (*EaCH*)’ cohort study primarily focuses on early developmental origins of unfavorable health outcomes through pre- and/or early postnatal exposure to a ‘diabetogenic/adipogenic’ environment. It includes 205 mother-child dyads, recruited between 2007 and 2010, from women with treated GDM and delivery at the Clinic of Obstetrics, Charité – Universitätsmedizin Berlin, Germany. Recruitment, therapy, metabolite/hormone analyses, and data evaluation were performed according to standardized guidelines and protocols. This report specifically aimed to identify maternal anthropometric and metabolic determinants of anthropometric and critical hormonal birth outcomes in ‘*EaCH’.*

**Results:**

Group comparisons, Spearman’s correlations and unadjusted linear regression analyses initially confirmed that increased maternal prepregnancy body-mass-index (BMI) is a significant factor for elevated birth weight, cord-blood insulin and leptin (all *P* < 0.05). However, consideration of and adjustment for maternal glucose during late pregnancy showed that no maternal anthropometric parameter (weight, BMI, gestational weight gain) remained significant (all n.s.). In contrast, even after adjustment for maternal anthropometrics, third trimester glucose values (fasting and postprandial glucose at 32nd and 36th weeks’ gestation, HbA1c in 3rd trimester and at delivery), were clearly positively associated with critical birth outcomes (all *P* < 0.05).

**Conclusions:**

Neither overweight/obesity nor gestational weight gain appear to be *independent* determinants of increased birth weight, insulin and leptin. Rather, 3rd trimester glycemia seems to be crucial for respective neonatal outcomes. Thus, gestational care and future research studies should greatly consider late pregnancy glucose in overweight/obese women with or without GDM, for evaluation of critical causes and interventional strategies against ‘perinatal programming of diabesity’ in the offspring.

## Background

Gestational diabetes mellitus (GDM) and maternal overweight/obesity are the most prevalent disorders/diseases during gestation, affecting > 10 and > 30% of pregnant women in westernized countries, respectively [1–4]. Both GDM as well as maternal overweight/obesity are causally linked to adverse birth and long-term outcomes in the offspring [5, 6]. Especially GDM, often a consequence of overweight/obesity, has manifold been identified epidemiologically, clinically and experimentally as risk condition in terms of critical birth outcomes related to long-term health adversity [7, 8]. In general, altered birth weight was recognized as an important surrogate marker for later disorders and diseases [9–11]. More precisely, altered humoral and hormonal parameters, e.g. increased glucose, insulin, leptin etc., in utero and at birth were identified as causal, mechanistic factors for ‘perinatal programming’ of increased susceptibility to develop disorders/diseases later in life [8, 12–14]. Similar ‘programming’ effects may also occur in offspring of overweight/obese mothers. Accordingly, during recent years overweight/obesity has been suggested, beyond GDM, as independent and additive risk condition for adverse short- and long-term outcomes [5, 15].

Fortunately, regular GDM screening and optimized treatment has become widely distributed since the past few years due to rapid progress in the GDM field. Moreover, greater emphasis worldwide has been placed on optimal management of glucose levels after a positive GDM screening test, especially as the HAPO study and large randomized controlled trials have strongly shown beneficial influences of treating GDM for birth outcomes [16–18]. However, similar convincing data are so far missing regarding the role of overweight during pregnancy. A major challenge here is the tight link between overweight/obesity and glycemia, which complicates the evaluation of the relative contribution of maternal anthropometry vs. glucose metabolism for birth outcomes. Moreover, most studies have solely relied on glucose values at GDM screening, typically performed at 24-28th weeks’ gestation, while maternal glycemia in later pregnancy is hardly considered. Thus, it remains open whether maternal overweight per se is an independent factor for respective adversity or if rather accompanied, undetected and/or not considered maternal (hyper-) glycemia, especially in 3rd trimester, is ultimately responsible. This needs further investigation since it would have far ranging implications for better understanding of pathophysiology, general risk estimation and optimal gestational care.

The 3rd trimester plays a major role for fetal growth, differentiation and maturation, in particular for growth of insulin-sensitive tissues, e.g. fat tissue [19]. Treatment of GDM, i.e., achieving good glycemic control, has been shown to effectively reduce in utero overgrowth and increased birth weight risk [17, 18]. Similarly, adequate glucose management has been observed to prevent perinatal hyperinsulinism and, consequently, related long-term risk of developing impaired glucose tolerance [20]. Interestingly, offspring of women with treated GDM may remain more likely to become overweight or exhibit features of the metabolic syndrome even with ‘normalized’ birth weight [21]. This indicates that ‘programming’ effects, which could similarly occur in offspring of overweight/obese mothers, are also apparent below adverse birth weight cut-offs, e.g. macrosomia. Exact mechanisms remain unknown but could be attributable to altered hormones during perinatal development, e.g. hyperinsulinism and hyperleptinism, even in newborns with ‘normal’ birth weight [8, 14]. Therefore, consideration of hormonal birth outcomes appears important in respective study interpretations.

In order to contribute to a clearer understanding of such important perinatal risk factors, the ‘Early CHARITÉ (*EaCH*)’ cohort was created to explore unreflected/unknown factors associated with the pre- and neonatal development in offspring of mothers with diabetes/overweight during pregnancy, and to characterize potential mechanisms and pathophysiological factors from the clinical to the molecular level. We initially focused on characterization of relations of maternal anthropometry, especially body-mass-index (BMI) and gestational weight gain (GWG), and maternal glucose metabolism on critical anthropometric and cord-blood hormone outcomes at birth.

## Methods

### Study aims and design

The *‘EaCH’* approach is a prospective observational cohort study in mother-child dyads with maternal GDM and/or overweight/obesity, with the primary focus on early developmental origins of unfavorable health outcomes through pre- and/or early postnatal exposure to a ‘diabetogenic/adipogenic’ environment. The primary aim of *‘EaCH’* is to identify metabolic, hormonal, nutritional, epigenetic and other causal factors/determinants that are critically associated with pre- and neonatal acquired disease susceptibility. The overall goal is to deliver new mechanistic and/or preventive approaches and strategies to reduce disease risk in affected offspring.

**Fig. 1:**
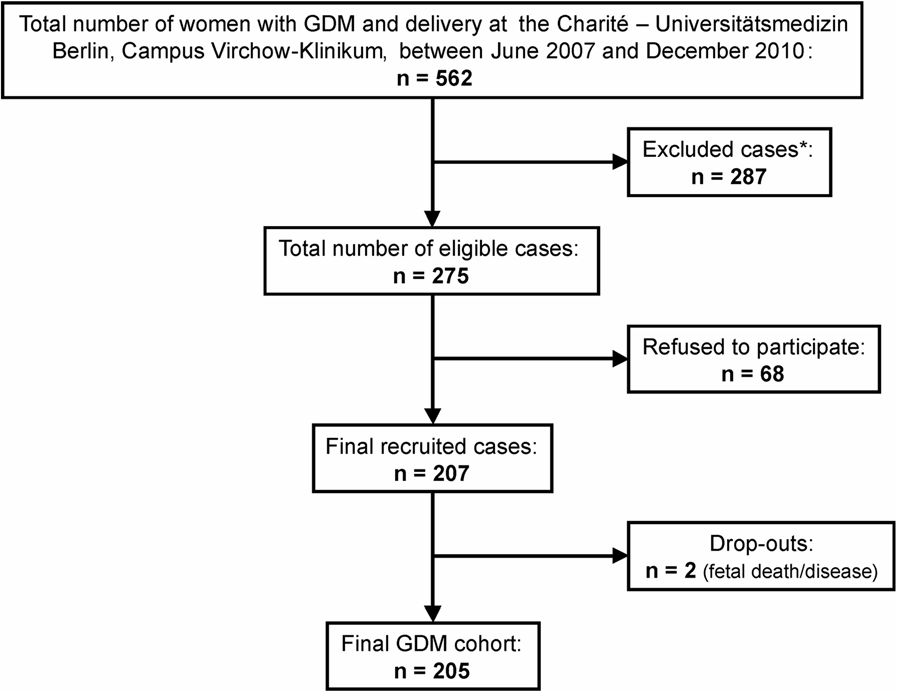
Flow chart of the *‘EaCH’* GDM cohort study population. *Exclusion criteria: missing German/English language skills, prepregnancy underweight (body-mass-index <18.5 kg/m^2^), assisted reproductive technology, chronic diseases/infections (including type 1 and type 2 diabetes; HIV, hepatitis B/C *etc.*), multiple pregnancy, substance abuse, special nutrition (*e.g.* vegetarians), special surgical conditions (*e.g. placenta* *previa*), first visit at the clinic after 32^nd^ weeks’ gestation and/or fetal diseases/malformations. GDM: Gestational diabetes mellitus

The study includes a final GDM cohort of 205 mother-child dyads (75% of eligible cases, Fig. 1), from initially 562 women with GDM and delivery between June 2007 and December 2010 at the Clinic of Obstetrics, Charité – Universitätsmedizin Berlin, Campus Virchow-Klinikum, Germany (Flow chart, including exclusion criteria, shown in Fig. 1). A major exclusion criterion was non-sufficient German/English speaking, due to a relatively high proportion of immigrants attending our clinic. Research design and methods were conducted in accordance with the Declaration of Helsinki, revised in 2004 [22], and approved by the local Ethics Committee (EA2/026/04). All participants provided written informed consent before inclusion into the study.

### Parental socio-economic, lifestyle, and anthropometric data

Respective data were collected at the woman’s first visit at our clinic by personal interview via standardized questionnaire. Parental educational background, current employment status (before maternity leave) and job description were recorded and used for socio-economic status (SES) categorization, as applied previously [23, 24]. The resulting SES categories were further assigned to lower (categories: unemployed, manual worker, non-manual worker without university degree) or higher SES (category: non-manual worker with university degree).

Paternal height and weight was recorded via standardized questionnaire at enrollment and the BMI (kg/m^2^) was calculated. Maternal height and weight before conception, weight change in the 1st, 2nd and 3rd trimester as well as the last weight measured within one week prior to delivery were abstracted/calculated from the ‘Mutterpass’. This ‘Mutterpass’ is a standardized maternity record/documentation in Germany, containing essential information about pregravid health/conditions, regular screening/medical examinations throughout pregnancy as well as clinically important perinatal data [25]. Prepregnancy BMI was calculated using pregravid weight and height and then categorized according to WHO criteria (normal weight: 18.5–24.9 kg/m^2^, overweight: 25.0–29.9 kg/m^2^, obese: ≥30.0 kg/m^2^). Total gestational weight gain (GWG) was calculated as the difference between prepregnancy weight and nearest weight to delivery and categorized in line with the Institute of Medicine (IOM) guidelines from 2009 using the labels inadequate, adequate and excessive weight gain, respectively [26]. As an indicator of the genuine maternal weight gain during pregnancy, net GWG was calculated by subtracting birth weight and placental weight from total GWG.

### GDM diagnosis and treatment

Gestational diabetes screening was conducted generally between the 24th–28th week of pregnancy (mean ± SD: 25.6 ± 3.8) using 75-g oral glucose tolerance test (oGTT) according to the guidelines of the German Society of Gynecology and Obstetrics at the time of recruitment [27]. Respective guidelines were based on the Carpenter and Coustan criteria [28]. GDM was diagnosed if two values were equal to or above either capillary fasting glucose (FG) ≥90 mg/dL (5.0 mmol/L) and/or 1-h glucose ≥180 mg/dL (10.0 mmol/L) and/or 2-h glucose ≥155 mg/dL (8.6 mmol/L). Subsequent diabetic care was provided according to above mentioned guidelines, aiming especially for the following glucose targets: FG 60–90 mg/dL (3.3–5.0 mmol/L) and 1-h postprandial glucose (PPG) ≤140 mg/dL (7.8 mmol/L). Among all GDM cases, 63% (*n* = 129) were treated by diet and moderate physical activity only, while 37% (*n* = 76) received additional insulin therapy to achieve glycemic control. Insulin-sensitizing drugs (e.g. metformin) were not applied here. Overall, women were instructed to monitor blood glucose at least 4-times daily, i.e., morning FG and three 1-h PPG values, using the Accu-Chek glucose meter (Roche Diagnostics, Mannheim, Germany). From these individual profiles, mean FG (based on three values) and mean PPG levels (based on nine values) were calculated from three days within the 32nd and 36th week of gestation, respectively. As marker for long-term glycemic status, glycated hemoglobin A1c (HbA1c) was assessed in the third trimester (mean ± SD: 33.2 ± 2.5 weeks’ gestation), and, additionally, at delivery by standardized high-performance liquid chromatography (Variant II System, Bio-Rad, Hercules, CA, USA) at the clinic’s central laboratory. Based on recent guidelines from the American Diabetes Association [29], an HbA1c value above 6% was used as indicator of poorer metabolic control for evaluations.

### Clinical pregnancy-related characteristics

A variety of maternal parameters, including parity, history of GDM, comorbidities (e.g. gestational hypertension), mode of delivery, gestational age at birth, were abstracted from the standardized ‘Mutterpass’ and medical records, respectively. Gestational hypertension and preeclampsia were diagnosed according to the guidelines of the German Society of Gynecology and Obstetrics [30]. Mode of delivery was defined according to HAPO study criteria [31], i.e., spontaneous, vaginal-operative, primary or repeat Cesarean section (CS). Gestational age was estimated based on woman’s last menstrual period in combination with subsequent ultrasound examinations at first trimester.

### Birth outcomes

Information about infant’s sex, preterm birth (< 37 weeks’ gestation), placental weight, birth weight, length and head circumference was abstracted from medical records. Placental weight was measured immediately after delivery with umbilical cord attached. Additional anthropometric outcomes included relative birth weight (g/cm), ponderal index (g/cm^3^ × 100), and macrosomia (defined as birth weight ≥ 4000 g). Furthermore, based on sex- and ethnic-specific birth weight for gestational age percentiles [32–34], infants were categorized into small-for-gestational age (SGA, <10th percentile), appropriate-for-gestational age (AGA, 10th–90th percentile) or large-for-gestational age (LGA, > 90th percentile). Occurrence of further adverse neonatal outcomes, e.g. hypoglycemia and/or shoulder dystocia, was retrieved from medical records.

Venous umbilical cord-blood was collected immediately after delivery and plasma samples stored at − 80 °C. Commercially available radioimmunoassays were used for analyses of insulin (Cat# 10624, Radim Diagnostics, Pomezia RM, Italy), C-peptide and leptin (C-peptide: Cat# RIA-1252, leptin: Cat# RIA-1624, DRG Instruments, Marburg, Germany) according to manufacturers’ instructions. The 90th percentile cut-offs in this study were the following: insulin 34.2 μU/mL, C-peptide 2.2 ng/mL, and leptin 29.6 ng/mL.

### Statistical analyses

Descriptive statistics are presented as means ± standard deviations (SD) or numbers and percentages, respectively. All continuous variables were tested for normal distribution using Kolmogorov-Smirnov test and evaluation of distribution plots. Group comparisons were analyzed by one-way ANOVA followed by Tukey’s post hoc test or Kruskal-Wallis test followed by Dunn’s post hoc test and Fisher’s exact or Chi-square tests, respectively. Spearman’s correlation coefficients (r) and/or linear regression models (unstandardized B coefficients with 95% confidence intervals [95% CI]) were calculated to assess relationships between paternal and/or maternal anthropometric/metabolic parameters and neonatal anthropometric and cord-blood hormone outcomes. For regression analyses, normal distribution was achieved for respective variables by transformation using the method described by Templeton [35]. Furthermore, categorical variables were dichotomized, e.g. mode of delivery was grouped into deliveries with vs. without CS etc., if required. Overall, collinearity was controlled by bivariate correlation (*r* < 0.7) and variance inflation factor (< 2.5). The following parameters were tested as independent variables for their association with birth outcomes. Anthropometry: maternal prepregnancy weight and BMI, GWG in 1st, 2nd and 3rd trimester, total and net GWG; Metabolism: FG, 1-h and 2-h glucose, and area under the curve (AUC) of glucose at oGTT, mean FG and PPG at 32nd and 36th weeks’ gestation, HbA1c in 3rd trimester and at delivery. Birth outcomes were: placental weight, birth weight, relative birth weight, cord-blood insulin and leptin. All regressions are shown as crude and adjusted models. Model I included general parameters: maternal age, SES, ethnic origin, smoking in pregnancy, parity, height (except for pre-pregnancy BMI), type of therapy (diet vs. insulin), gestational age, mode of delivery, and infant’s sex. To evaluate independent associations of maternal anthropometry and metabolism with birth outcomes, further adjustment models were applied. For maternal anthropometry, model II contained all parameters of model I *plus* mean FG and PPG at 32nd and 36th weeks’ gestation and HbA1c partum. Accordingly, the applied model II for maternal metabolism included all parameters of model I *plus* prepregnancy weight and net GWG. In addition, forward step-wise regression analyses were carried out to identify categorical predictors of anthropometric/hormonal birth outcomes, considering paternal and maternal parameters (max. *n* = 118–153). In line with previous studies in this research area [5, 36], adjusted semipartial correlation coefficients (Sr^2^) were calculated for each individual (potential) predictor entered into the final models. These coefficients estimate the contribution of each factor to the total variability of the outcome variable. Finally, owing to small numbers of unfavorable categorical anthropometric and cord-blood hormone outcomes logistic regression analyses (odds ratio, OR) were just performed for LGA (*n* = 41 in the total cohort), using the same adjustment models as described above. Additionally, percentages of relevant maternal anthropometric and metabolic variables/exposures in newborns with unfavorable anthropometric and cord-blood hormone outcomes were compared with those in reference groups that had no respective outcomes. In general, missing data analysis according to Little’s MCAR test was performed and indicated random distribution. A *P*-value < 0.05 was considered significant (two-tailed). All calculations were performed using SPSS v. 24.0 (IBM Co., Armonk, NY, USA).

## Results

### General parental characteristics

Major parameters across maternal prepregnancy BMI categories are presented in Table 1. Overall, the mean paternal BMI as well as paternal SES were in alignment with those of the mothers, with highest BMI and lowest SES in partners of obese mothers (Table 1). Moreover, there was a positive correlation between paternal and maternal BMIs (*r* = 0.23, *P* < 0.01).

**Table 1:** General and specific cohort characteristics

Characteristics	Total GDM cohort		Maternal prepregnancy BMI category			
			Normal weight	Overweight	Obese	*P*-value^a^
	*N*					
n		205	89^b^	51^b^	65^b^	
Paternal						
Age (years)	204	35.7 ± 7.7	35.9 ± 7.2	36.2 ± 7.6	34.9 ± 8.4	ns
Socio-economic status – %^c^						
Lower SES category	143	69.8	54.5	68.0	93.8^d^	< 0.001
Higher SES category	60	29.3	45.5	32.0	6.2^d^	< 0.001
BMI (kg/m^2^)	202	26.9 ± 5.0	25.5 ± 3.2	27.0 ± 4.7^d^	28.7 ± 6.4^d^	0.002
Maternal						
Age (years)	205	31.8 ± 5.4	32.2 ± 5.5	31.2 ± 4.9	31.7 ± 5.8	ns
Ethnic origin – %						
European	129	62.9	73.0	52.9^d^	56.9^d^	< 0.001
Non-European (e.g. Turkish, Arabic)	76	37.1	27.0	47.1^d^	43.1^d^	< 0.001
Socio-economic status – %^c^						
Lower SES category	161	78.5	62.9	86.3^d^	93.8^d^	< 0.001
Higher SES category	44	21.1	37.1	13.7^d^	6.2^d^	< 0.001
Smoking in pregnancy (any) – %	30	14.6	10.1	7.8	26.2^d^	0.009
Nulliparous – %	61	29.8	37.1	37.3	13.9^d^	0.002
History of GDM (multiparous only) – %	52	40.6	29.2	48.4	46.9	ns
Height (cm)	205	165.8 ± 6.6	166.9 ± 6.6	164.9 ± 7.0	165.1 ± 6.2	ns
Prepregnancy weight (kg)	205	76.8 ± 20.9	60.6 ± 6.7	73.8 ± 6.6^d^	101.4 ± 17.6^d^	< 0.001
Prepregnancy BMI (kg/m^2^)	205	28.0 ± 7.4	21.7 ± 1.7	27.1 ± 1.4^d^	37.1 ± 5.5^d^	< 0.001
Total GWG (kg)	205	13.4 ± 6.4	14.8 ± 5.3	14.0 ± 7.1	10.8 ± 6.4^d^	< 0.001
GWG category (IOM) – %						
Inadequate	43	21.0	28.1	19.6	12.3^d^	ns
Adequate	67	32.7	37.1	23.5	33.9	ns
Excessive	95	46.3	34.8	56.9^d^	53.8^d^	0.014
Net GWG (kg)	205	9.3 ± 6.3	11.0 ± 5.2	9.7 ± 7.0	6.6 ± 6.3^d^	< 0.001
BMI at delivery (kg/m^2^)	205	32.8 ± 7.1	27.1 ± 2.5	32.3 ± 2.7^d^	41.1 ± 5.5^d^	< 0.001
Blood glucose at oGTT (mg/dL)^e^						
Fasting	205	94.2 ± 19.0	88.6 ± 13.3	91.8 ± 14.3	103.7 ± 24.7^d^	< 0.001
Fasting > 90 mg/dL – %^f^	118	57.6	42.7	54.9	80.0^d^	< 0.001
1 h	203	197.0 ± 28.2	196.0 ± 26.0	192.8 ± 24.0	201.8 ± 33.4	ns
1 h > 180 mg/dL – %^f^	169	83.7	86.5	80.0	82.5	ns
2 h	193	144.5 ± 37.6	140.0 ± 27.3	143.3 ± 32.4	151.4 ± 50.6	ns
2 h > 155 mg/dL – %^f^	63	32.8	28.6	34.0	37.7	ns
Area under the curve (mg/dL*h)	193	315.6 ± 44.3	310.2 ± 32.9	306.9 ± 31.0	329.6 ± 60.6	ns
Mean glucose at 32nd weeks’ gestation (mg/dL)^g^						
Fasting	195	88.0 ± 11.3	83.8 ± 7.0	87.8 ± 9.0^d^	93.8 ± 14.5^d^	< 0.001
Fasting > 90 mg/dL – %^f^	70	35.9	16.9	34.7^d^	61.9^d^	< 0.001
Postprandial	183	121.7 ± 14.3	117.6 ± 11.5	122.8 ± 13.3^d^	126.4 ± 16.8^d^	< 0.001
Postprandial > 140 mg/dL – %^f^	14	7.7	2.5	6.8	15.0^d^	0.020
Mean glucose at 36th weeks’ gestation (mg/dL)^g^						
Fasting	188	84.2 ± 8.0	80.8 ± 6.0	85.2 ± 8.0^d^	87.7 ± 8.4^d^	< 0.001
Fasting > 90 mg/dL – %^f^	38	20.2	6.3	20.0^d^	37.5^d^	< 0.001
Postprandial	169	118.6 ± 12.1	114.0 ± 8.9	121.5 ± 14.0^d^	122.3 ± 12.2^d^	ns
Postprandial > 140 mg/dL – %^f^	6	3.6	0.0	4.8	7.1^d^	ns
HbA1c in 3rd trimester (%)	196	5.4 ± 0.6	5.1 ± 0.4	5.5 ± 0.6^d^	5.7 ± 0.6^d^	< 0.001
HbA1c in 3rd trimester > 6% – %	24	12.2	2.4	18.8^d^	20.3^d^	< 0.001
HbA1c partum (%)	197	5.5 ± 0.6	5.3 ± 0.5	5.6 ± 0.5^d^	5.8 ± 0.6^d^	< 0.001
HbA1c partum > 6% – %	42	21.3	6.9	23.9^d^	39.1^d^	< 0.001
Insulin therapy – %	75	36.6	16.9	41.2^d^	60.0^d^	< 0.001
Prepregnancy hypertension – %	10	4.9	2.2	2.0	10.8^d^	0.035
Gestational hypertension – %	12	5.9	2.2	5.9	10.8^d^	ns
Preeclampsia – %	9	4.4	2.2	2.0	9.2	ns
Mode of delivery – %						
Spontaneous	115	56.1	66.3	45.1^d^	50.8	0.028
Vaginal-operative	20	9.8	7.9	17.7	6.2	ns
Primary Cesarean section	31	15.1	15.7	11.8	16.9	ns
Repeat Cesarean section	39	19.0	10.1	25.5^d^	26.2^d^	0.015
Gestational age at delivery (weeks)	205	38.6 ± 1.5	38.7 ± 1.6	38.9 ± 1.3	38.3 ± 1.5^d^	ns
Newborn						
Female sex – %	88	42.9	43.8	43.1	41.5	ns
Preterm birth (< 37 weeks’ gestation) – %	15	7.3	6.7	3.9	10.8	ns
Placental weight (g)	201	607.0 ± 159.9	559.4 ± 124.6	666.1 ± 172.3^d^	624.9 ± 175.1^d^	< 0.001
Birth weight (g)	205	3450 ± 506	3345 ± 496	3591 ± 481^d^	3483 ± 514	0.006
Birth length (cm)	205	51.3 ± 2.5	51.2 ± 2.5	51.7 ± 2.4	50.9 ± 2.5	ns
Relative birth weight (g/cm)	205	67.1 ± 8.1	65.1 ± 7.8	69.3 ± 7.7^d^	68.2 ± 8.4^d^	0.001
Ponderal index (g/cm^3^ × 100)	205	2.6 ± 0.3	2.5 ± 0.2	2.6 ± 0.3	2.6 ± 0.3^d^	0.009
Birth weight category – %						
SGA	9	4.4	4.5	2.0	6.2	ns
AGA	155	75.6	84.3	70.6	67.7^d^	0.036
LGA	41	20.0	11.2	27.5^d^	26.2^d^	0.018
Macrosomia (≥4000 g) – %	27	13.2	7.9	21.6^d^	13.8	ns
Head circumference (cm)	205	34.8 ± 2.9	34.7 ± 1.8	35.3 ± 1.4	35.0 ± 1.5	ns
Hypoglycemia – %	20	9.8	5.6	7.8	16.9^d^	ns
Shoulder dystocia – %	1	0.5	0.0	0.0	1.5	ns
Cord-blood plasma levels						
C-peptide (ng/mL)	162	1.4 ± 0.7	1.3 ± 0.6	1.4 ± 0.7	1.6 ± 0.7^d^	0.016
C-peptide > 90th percentile – %^h^	17	10.5	5.4	7.7	20.4^d^	0.034
Insulin (μU/mL)	164	17.3 ± 13.7	14.3 ± 10.6	18.5 ± 17.6	20.7 ± 13.6^d^	0.022
Insulin > 90th percentile – %^h^	15	9.2	5.4	7.9	15.7	ns
Leptin (ng/mL)	164	13.3 ± 10.7	10.6 ± 8.6	15.2 ± 11.1^d^	15.9 ± 12.3^d^	0.013
Leptin > 90th percentile – %^h^	16	9.8	6.8	10.3	13.7	ns

Data are shown as mean ± SD or %. *N*: Number of subjects with characteristic. n: Total number of subjects

Abbreviations: *GDM* Gestational diabetes mellitus, *BMI* Body-mass-index, *ns* Non-significant, *SES* Socio-economic status, *GWG* Gestational weight gain, *IOM* Institute of Medicine, *oGTT* Oral glucose tolerance test, *HbA1c* Glycated hemoglobin A1c, *SGA* Small-for-gestational age, *AGA* Appropriate-for-gestational age, *LGA* Large-for-gestational age

^a^Statistical significant differences (*P* < 0.05) across all pregestational BMI groups were assessed using one-way ANOVA with Tukey’s post hoc test or Kruskal-Wallis test with Dunn’s post hoc test and Fisher’s exact or Chi-squared tests, as appropriate. ^b^n across variables: Normal weight = 74–89, overweight = 39–51, obese = 49–65. ^c^Lower (unemployed, manual and non-manual worker without university degree) or higher SES (non-manual worker with university degree) [23, 24]. ^d^Statistical significant difference vs. normal weight. ^e^oGTT at 25.6 ± 3.8 weeks’ gestation. ^f^Blood glucose values above guideline cut-offs at the time of study [27]. ^g^Mean fasting and 1-h postprandial glucose values were calculated using self-monitored blood glucose data from three days at respective weeks’ gestation. ^h^Study 90th percentile cut-offs: C-Peptide 2.2 ng/mL, Insulin 34.2 μU/mL, Leptin 29.6 ng/mL.

Maternal data showed no difference in age across prepregnancy BMI groups (Table 1). Overall, the majority of women of the total cohort was of European origin and belonged to the lower SES category, while significantly more individuals in the overweight/obese groups were of Non-European origin and had higher frequencies of lower SES. There was no difference in smoking during pregnancy between normal weight and overweight (but not obese) women; however, smoking was much more abundant in the obese group. Similarly, parity was higher in obese individuals. Amongst multiparous women, more than one-third had a history of GDM, while a non-significant trend towards higher frequencies of prior GDM was observed in overweight/obese groups compared to normal weight mothers.

### Maternal anthropometrics

The GDM cohort as a whole was overweight, in terms of mean BMI, before pregnancy. Around 40% were classified as normal weight and around 30% as obese. More than half of the women in the overweight/obese groups showed excessive GWG, according to IOM criteria, as compared to only around one-third in normal weight women. Furthermore, there was a continuous decrease in inadequate GWG across prepregnancy BMI groups. Total GWG was lowest in obese women, while normal weight and overweight mothers gained similar amounts of weight during gestation. However, the calculated net GWG, i.e., excluding placental and newborn weight, showed a decrease across all BMI categories (Table 1). Both total and net GWG correlated inversely with prepregnancy BMI (total GWG: *r* = − 0.30, *P* < 0.001; net GWG: *r* = − 0.32, *P* < 0.001).

### Maternal metabolism

In the total cohort, GDM screening revealed that the majority of women exceeded FG and 1-h glucose cut-offs while only one-third surpassed the 2-h glucose threshold at oGTT (Table 1). Among oGTT measurements, only FG and frequencies above its cut-off showed significant increase across BMI categories. In 3rd trimester, an overall decrease of mean FG and PPG values, and, thus, a reduction of frequencies above their respective guideline cut-offs was observed. Of note, application of guideline cut-offs (FG > 90 mg/dL; PPG > 140 mg/dL) regarding 3rd trimester values revealed that to a greater extent FG rather than PPG was less controlled. In line with this, among all glucose values at oGTT, only FG correlated significantly with the respective mean glucose levels later in pregnancy (FG at 32nd weeks: *r* = 0.40, *P* < 0.001; FG at 36th weeks: *r* = 0.27, *P* < 0.001). Compared to normal weight women, insulin therapy was more abundant in the overweight and obese groups, respectively. Each glucose variable in 3rd trimester, i.e., mean glucose and HbA1c levels, increased significantly with maternal prepregnancy BMI, in contrast to oGTT variables at 24–28 weeks’ gestation (Table 1).

### Birth outcomes

Placental weight was significantly higher in the overweight/obese groups as compared to normal weight women (Table 1). Likewise, birth weight, relative birth weight and ponderal index were higher in overweight/obese individuals. However, instead of a further increase in the obese as compared to overweight women these anthropometric measures plateaued. Moreover, birth weight and relative birth weight were highest in the overweight group. Frequencies of adverse anthropometric outcomes were more abundant in the overweight/obese groups, with the exception of SGA as there was no significant difference across BMI categories. Overweight women had the highest rate of LGA and macrosomia. In obese, the percentage of LGA was similar to the overweight group, however, the rate of macrosomia was between the normal weight and overweight category. In contrast to these observations regarding anthropometric birth outcomes, cord-blood levels of C-peptide, insulin, and leptin continuously increased across BMI categories. Accordingly, the same pattern was observed for frequencies above the 90th percentile cut-offs of these hormones at birth.

### Associations between parental anthropometric and maternal metabolic variables and birth outcomes

Paternal BMI showed no relation to *any* of the investigated birth outcomes, neither in step-wise regression (see below) nor correlation analyses (Spearman’s r of paternal BMI: vs. birth weight, *r* = − 0.01; vs. relative birth weight, *r* = 0.02; vs. cord-blood insulin, *r* = 0.13; vs. cord-blood leptin, *r* = − 0.02; all not significant).

Bivariate associations between maternal anthropometry and glycemia revealed that prepregnancy BMI positively correlated only with FG among oGTT measurements (*r* = 0.36, *P* < 0.001) but clearly with 3rd trimester glucose variables (mean FG at 32nd weeks’ gestation: *r* = 0.40, mean FG at 36th weeks’ gestation: *r* = 0.41, HbA1c in 3rd trimester: *r* = 0.50, HbA1c at delivery: *r* = 0.46; all *P* < 0.001). In contrast, the only relationship of net GWG was an inverse correlation with AUC glucose at oGTT (*r* = − 0.15, *P* = 0.037).

**Fig. 2:**
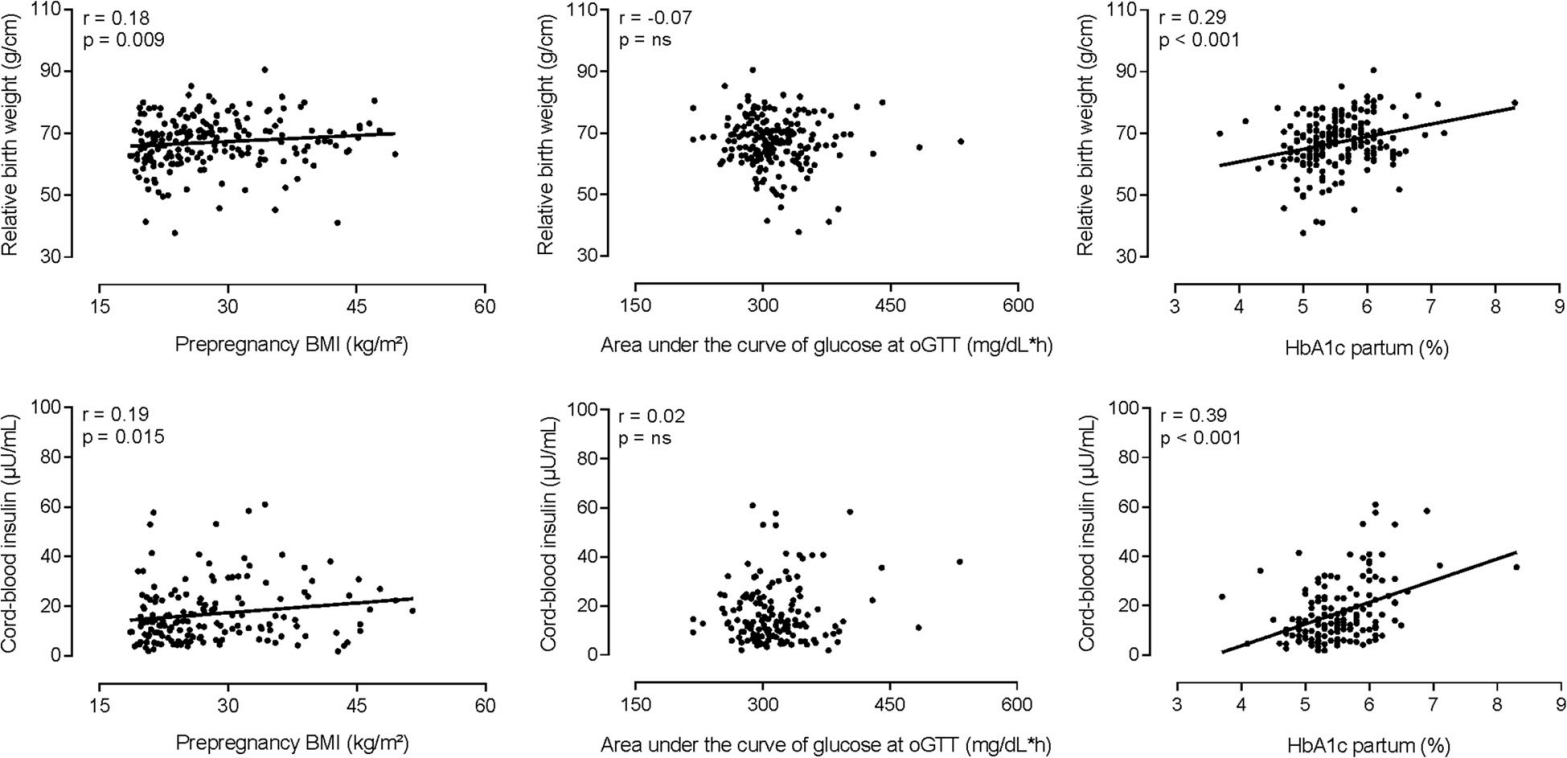
Correlations between maternal parameters and birth outcomes. Maternal prepregnancy body-mass-index (BMI) and glycemia at delivery (HbA1c), but not at oral glucose tolerance test (oGTT), were significantly positively associated with relative birth weight and cord-blood insulin levels. Spearman’s correlation coefficients (r) and *P*-values are shown

Furthermore, prepregnancy BMI, but not net GWG, was significantly positively associated with birth outcomes, i.e., placental weight, relative birth weight, cord-blood insulin and leptin (Table 2, Fig. 2). The relationship between prepregnancy BMI and birth weight was close to significance (*r* = 0.14, *P* = 0.050). Glucose variables at oGTT were not related to birth outcomes, with the exception of a weak positive correlation between FG and placental weight. However, mean FG at 32nd weeks’ gestation and HbA1c levels in 3rd trimester and at delivery were significantly positively associated with all birth outcomes, including birth weight (Table 2). Among all anthropometric and metabolic variables, HbA1c at delivery had the strongest associations with birth outcomes, in particular with relative birth weight and cord-blood insulin (Table 2, Fig. 2). Of note, placental weight was an even stronger correlate for birth weight (*r* = 0.57, *P* < 0.001) and relative birth weight (*r* = 0.58, *P* < 0.001).

**Table 2:** Spearman’s correlations between key maternal anthropometric and metabolic variables and neonatal outcomes

Maternal characteristics	Neonatal outcomes									
	Placental weight		Birth weight		Relative birth weight		Cord-blood insulin		Cord-blood leptin	
	r	*P*-value	r	*P*-value	r	*P*-value	r	*P*-value	r	*P*-value
Prepregnancy BMI	0.24	0.001	0.14	ns	0.18	0.009	0.19	0.015	0.20	0.009
Net GWG	0.06	ns	0.09	ns	0.09	ns	0.04	ns	− 0.06	ns
FG at oGTT	0.18	0.010	0.12	ns	0.13	ns	0.10	ns	0.15	ns
AUC glucose at oGTT	−0.02	ns	− 0.09	ns	− 0.07	ns	0.02	ns	0.15	ns
Mean FG at 32nd GW	0.24	< 0.001	0.16	0.029	0.19	0.007	0.23	0.003	0.23	0.004
Mean FG at 36th GW	0.12	ns	0.10	ns	0.15	0.041	0.21	0.010	0.21	0.011
HbA1c in 3rd trimester	0.21	0.004	0.17	0.017	0.22	0.002	0.31	< 0.001	0.32	< 0.001
HbA1c partum	0.24	< 0.001	0.25	< 0.001	0.29	< 0.001	0.39	< 0.001	0.38	< 0.001

Abbreviations: *BMI* Body-mass-index, *ns* Non-significant, *GWG* Gestational weight gain, *FG* Fasting glucose, *oGTT* Oral glucose tolerance test, *AUC* Area under the curve, *GW* Gestational week, *HbA1c* Glycated hemoglobin A1c

The influence of maternal anthropometry on birth outcomes was further investigated in detail by comprehensive regression analyses (Table 3). There were no relationships observed between GWG in 1st and 2nd trimester and net GWG, respectively, and anthropometric birth outcomes. In the crude models, prepregnancy weight status, i.e., body weight and/or BMI, and 3rd trimester and/or total GWG were significantly positively associated with placental weight, birth weight and relative birth weight. However, their initial relations were already diminished after adjustment to general parameters (Model I, Table 3). Further addition of maternal 3rd trimester glucose variables (Model II, Table 3) completely abolished all of these associations. With regard to cord-blood hormones, there were hardly any associations observed with maternal anthropometry. In unadjusted models, only prepregnancy weight and BMI status showed significant positive associations with insulin and leptin, however, these relations no longer persisted even after adjustment only to general variables (Model I, Table 3). Taken together, neither maternal nor paternal anthropometry showed a significant relation to investigated birth outcomes, after consideration of maternal metabolic parameters.

**Table 3:** Crude and adjusted linear regression analyses for maternal anthropometric variables vs. neonatal anthropometric and cord-blood outcome variables

Outcome variable	Independent variable	Crude		Adjusted^a^			
				Model I		Model II	
		B (95% CI)	*P*-value	B (95% CI)	*P*-value	B (95% CI)	*P*-value
Placental weight							
	Prepregnancy body weight	0.195 (0.06; 0.34)	0.006	0.171 (0.02; 0.33)	0.032	0.118 (− 0.09; 0.32)	ns
	Prepregnancy BMI	0.201 (0.06; 0.34)	0.005	0.164 (0.01; 0.32)	0.036	0.097 (− 0.10; 0.30)	ns
	GWG in 1. trimester	0.066 (− 0.08; 0.21)	ns	0.077 (−0.06; 0.21)	ns	0.052 (− 0.10; 0.20)	ns
	GWG in 2. trimester	0.082 (− 0.06; 0.22)	ns	0.058 (− 0.09; 0.20)	ns	0.029 (− 0.14; 0.19)	ns
	GWG in 3. trimester	0.184 (0.04; 0.32)	0.010	0.131 (−0.01; 0.27)	ns	0.057 (− 0.10; 0.22)	ns
	Total GWG	0.120 (−0.02; 0.26)	ns	0.099 (−0.04; 0.24)	ns	0.018 (− 0.14; 0.18)	ns
	Net GWG	0.054 (−0.09; 0.20)	ns	0.045 (−0.09; 0.18)	ns	−0.034 (− 0.19; 0.12)	ns
Birth weight							
	Prepregnancy body weight	0.142 (0.00; 0.28)	0.044	0.113 (− 0.03; 0.25)	ns	0.085 (−0.10; 0.27)	ns
	Prepregnancy BMI	0.103 (− 0.04; 0.24)	ns	0.102 (−0.04; 0.24)	ns	0.081 (−0.11; 0.27)	ns
	GWG in 1. trimester	0.043 (−0.10; 0.18)	ns	0.058 (−0.06; 0.18)	ns	0.025 (−0.11; 0.17)	ns
	GWG in 2. trimester	0.139 (− 0.00; 0.28)	ns	0.084 (−0.04; 0.21)	ns	0.091 (−0.06; 0.24)	ns
	GWG in 3. trimester	0.190 (0.05; 0.33)	0.007	0.146 (0.02; 0.27)	0.021	0.113 (− 0.03; 0.26)	ns
	Total GWG	0.172 (0.03; 0.31)	0.016	0.139 (0.02; 0.26)	0.028	0.108 (−0.04; 0.25)	ns
	Net GWG	0.086 (− 0.05; 0.23)	ns	0.077 (−0.05; 0.20)	ns	0.048 (−0.10; 0.19)	ns
Relative birth weight							
	Prepregnancy body weight	0.188 (0.05; 0.33)	0.007	0.133 (− 0.01; 0.28)	ns	0.069 (− 0.12; 0.26)	ns
	Prepregnancy BMI	0.150 (0.01; 0.29)	0.033	0.121 (−0.02; 0.26)	ns	0.069 (− 0.12; 0.26)	ns
	GWG in 1. trimester	0.026 (−0.12; 0.17)	ns	0.038 (− 0.09; 0.16)	ns	0.043 (− 0.10; 0.19)	ns
	GWG in 2. trimester	0.136 (−0.01; 0.28)	ns	0.105 (−0.03; 0.24)	ns	0.107 (−0.05; 0.26)	ns
	GWG in 3. trimester	0.216 (0.08; 0.35)	0.002	0.171 (0.05; 0.30)	0.008	0.105 (−0.04; 0.25)	ns
	Total GWG	0.197 (0.06; 0.33)	0.005	0.168 (0.04; 0.29)	0.009	0.131 (−0.02; 0.28)	ns
	Net GWG	0.106 (− 0.03; 0.25)	ns	0.098 (−0.03; 0.23)	ns	0.064 (−0.08; 0.21)	ns
Cord-blood insulin							
	Prepregnancy body weight	0.109 (−0.04; 0.26)	ns	0.078 (−0.09; 0.24)	ns	−0.046 (− 0.25; 0.16)	ns
	Prepregnancy BMI	0.177 (0.03; 0.33)	0.023	0.090 (−0.07; 0.25)	ns	−0.021 (− 0.23; 0.18)	ns
	GWG in 1. trimester	−0.067 (− 0.22; 0.09)	ns	− 0.059 (− 0.20; 0.08)	ns	− 0.109 (− 0.26; 0.05)	ns
	GWG in 2. trimester	0.050 (− 0.11; 0.21)	ns	0.083 (− 0.07; 0.24)	ns	0.157 (− 0.01; 0.32)	ns
	GWG in 3. trimester	0.142 (− 0.01; 0.30)	ns	0.111 (− 0.03; 0.26)	ns	0.146 (−0.02; 0.31)	ns
	Total GWG	0.107 (−0.05; 0.26)	ns	0.129 (−0.01; 0.27)	ns	0.147 (−0.01; 0.31)	ns
	Net GWG	0.069 (−0.09; 0.23)	ns	0.102 (−0.04; 0.25)	ns	0.124 (−0.04; 0.28)	ns
Cord-blood leptin							
	Prepregnancy body weight	0.159 (0.01; 0.31)	0.042	0.116 (− 0.05; 0.28)	ns	0.102 (−0.11; 0.31)	ns
	Prepregnancy BMI	0.177 (0.02; 0.33)	0.024	0.098 (− 0.06; 0.26)	ns	0.088 (− 0.12; 0.29)	ns
	GWG in 1. trimester	−0.032 (− 0.19; 0.13)	ns	− 0.018 (− 0.16; 0.12)	ns	− 0.061 (− 0.22; 0.10)	ns
	GWG in 2. trimester	−0.102 (− 0.26; 0.06)	ns	− 0.049 (− 0.20; 0.10)	ns	0.031 (− 0.14; 0.20)	ns
	GWG in 3. trimester	0.117 (− 0.04; 0.27)	ns	0.069 (−0.08; 0.22)	ns	0.103 (− 0.06; 0.27)	ns
	Total GWG	0.006 (−0.15; 0.16)	ns	0.032 (−0.11; 0.18)	ns	0.022 (− 0.14; 0.18)	ns
	Net GWG	−0.019 (− 0.18; 0.14)	ns	0.020 (− 0.12; 0.16)	ns	0.015 (− 0.15; 0.18)	ns

Abbreviations: *BMI* Body-mass-index, *ns* Non-significant, *GWG* Gestational weight gain

^a^Model I: Adjusted for maternal age, socio-economic status, ethnic origin, smoking during pregnancy, parity, height (except for BMI), type of therapy, gestational age, mode of delivery and infant’s sex.

Model II: Model I adjustment plus mean fasting and postprandial glucose at 32nd and 36th weeks’ gestation and HbA1c partum

In order to identify potential metabolic determinants responsible for these observations, regression analyses with a variety of glucose variables were carried out (Table 4). Glucose values at oGTT showed almost no relationship with any birth outcome. There was just a positive relation between FG at oGTT and placental weight, as already observed in correlation analysis (Table 2). In contrast, even an inverse relation between 1-h glucose and birth weight was observed (Table 4). Both associations, however, were no longer present after adjustment to general variables (Model I, Table 4). On the contrary, 3rd trimester glucose variables were significantly positively associated with anthropometric outcomes at birth. Particularly consistent, mean FG and PPG at 32nd weeks’ gestation as well as HbA1c levels in 3rd trimester and at delivery were significantly positively associated with all anthropometric outcomes in the crude models. Importantly, most of these relations remained constant also after adjustment to general parameters (Model I, Table 4). Further inclusion of maternal anthropometric variables into the adjustment model (Model II, Table 4) abolished some initial associations. However, after adjustment to critical general as well as anthropometric variables (Model II) mean glucose values at 32nd and 36th weeks’ gestation showed clear significant positive relationships with placental weight, while mean PPG at 32nd weeks’ gestation and HbA1c in 3rd trimester and at delivery were significantly positively associated with birth weight and relative birth weight, respectively (Table 4). A similar pattern was observed for critical cord-blood hormones. Again, glucose variables at oGTT were hardly related to cord-blood insulin and leptin. The only association was observed between FG at oGTT and cord-blood leptin, however, this weak positive relationship was only present in the unadjusted model. In contrast, considering 3rd trimester glucose variables, in particular mean FG at 32nd and 36th weeks’ gestation and HbA1c at delivery remained significantly positively associated with cord-blood insulin, even after full adjustment (Model II, Table 4). Likewise, maternal 3rd trimester glycemia, indicated by HbA1c, was significantly positively related to cord-blood leptin, and HbA1c at delivery showed the strongest effects here (Table 4). Adjustment to general and, additionally, maternal anthropometric variables only marginally altered the effect size of these associations (Model II, Table 4).

**Table 4:** Crude and adjusted linear regression analyses for maternal metabolic variables vs. neonatal anthropometric and cord-blood outcome variables

Outcome variable	Independent variable	Crude		Adjusted^a^			
				Model I		Model II	
		B (95% CI)	*P*-value	B (95% CI)	*P*-value	B (95% CI)	*P*-value
Placental weight							
	FG at oGTT	0.170 (0.03; 0.31)	0.017	0.106 (−0.04; 0.25)	ns	0.072 (− 0.08; 0.22)	ns
	1-h glucose at oGTT	−0.084 (− 0.23; 0.06)	ns	− 0.088 (− 0.23; 0.05)	ns	−0.051 (− 0.19; 0.09)	ns
	2-h glucose at oGTT	0.046 (−0.10; 0.19)	ns	0.065 (−0.08; 0.21)	ns	0.080 (−0.07; 0.23)	ns
	AUC glucose at oGTT	−0.034 (− 0.18; 0.11)	ns	− 0.037 (− 0.19; 0.11)	ns	−0.008 (− 0.16; 0.14)	ns
	Mean FG at 32nd GW	0.250 (0.11; 0.39)	0.001	0.231 (0.07; 0.39)	0.006	0.197 (0.03; 0.36)	0.021
	Mean PPG at 32nd GW	0.300 (0.16; 0.44)	< 0.001	0.265 (0.11; 0.42)	0.001	0.234 (0.08; 0.39)	0.003
	Mean FG at 36th GW	0.145 (−0.00; 0.29)	ns	0.125 (− 0.03; 0.28)	ns	0.071 (− 0.09; 0.23)	ns
	Mean PPG at 36th GW	0.218 (0.07; 0.37)	0.005	0.215 (0.06; 0.37)	0.008	0.179 (0.02; 0.34)	0.040
	HbA1c in 3.trimester	0.220 (0.08; 0.36)	0.003	0.192 (0.04; 0.35)	0.015	0.141 (−0.02; 0.31)	ns
	HbA1c partum	0.222 (0.08; 0.37)	0.003	0.151 (−0.00; 0.30)	ns	0.106 (−0.05; 0.27)	ns
Birth weight							
	FG at oGTT	0.094 (−0.05; 0.23)	ns	0.052 (−0.08; 0.19)	ns	0.062 (− 0.08; 0.20)	ns
	1-h glucose at oGTT	−0.142 (− 0.28; − 0.00)	0.044	−0.091 (− 0.22; 0.04)	ns	−0.059 (− 0.19; 0.07)	ns
	2-h glucose at oGTT	−0.049 (− 0.19; 0.09)	ns	0.016 (− 0.12; 0.15)	ns	0.045 (− 0.09; 0.18)	ns
	AUC glucose at oGTT	−0.126 (− 0.27; 0.02)	ns	− 0.057 (− 0.19; 0.08)	ns	− 0.014 (− 0.15; 0.12)	ns
	Mean FG at 32nd GW	0.157 (0.01; 0.30)	0.032	0.147 (− 0.00; 0.30)	ns	0.120 (−0.03; 0.27)	ns
	Mean PPG at 32nd GW	0.295 (0.15; 0.44)	< 0.001	0.259 (0.12; 0.40)	< 0.001	0.233 (0.09; 0.38)	0.002
	Mean FG at 36th GW	0.125 (−0.02; 0.27)	ns	0.118 (−0.02; 0.26)	ns	0.080 (−0.06; 0.22)	ns
	Mean PPG at 36th GW	0.161 (0.01; 0.31)	0.033	0.171 (0.03; 0.32)	0.020	0.111 (−0.04; 0.26)	ns
	HbA1c in 3.trimester	0.141 (0.00; 0.28)	0.048	0.146 (0.01; 0.29)	0.040	0.112 (−0.04; 0.26)	ns
	HbA1c partum	0.218 (0.08; 0.36)	0.003	0.186 (0.05; 0.32)	0.008	0.176 (0.03; 0.32)	0.016
Relative birth weight							
	FG at oGTT	0.108 (−0.03; 0.25)	ns	0.031 (− 0.11; 0.17)	ns	0.034 (− 0.11; 0.17)	ns
	1-h glucose at oGTT	−0.108 (− 0.25; 0.03)	ns	− 0.074 (− 0.20; 0.06)	ns	−0.046 (− 0.18; 0.09)	ns
	2-h glucose at oGTT	0.005 (−0.14; 0.15)	ns	0.050 (−0.09; 0.18)	ns	0.088 (−0.05; 0.22)	ns
	AUC glucose at oGTT	−0.080 (− 0.22; 0.06)	ns	− 0.039 (− 0.18; 0.10)	ns	0.003 (− 0.14; 0.14)	ns
	Mean FG at 32nd GW	0.193 (0.05; 0.34)	0.008	0.142 (−0.01; 0.29)	ns	0.105 (−0.05; 0.26)	ns
	Mean PPG at 32nd GW	0.319 (0.18; 0.46)	< 0.001	0.285 (0.14; 0.43)	< 0.001	0.241 (0.09; 0.39)	0.002
	Mean FG at 36th GW	0.174 (0.03; 0.32)	0.017	0.139 (−0.00; 0.28)	ns	0.098 (−0.05; 0.24)	ns
	Mean PPG at 36th GW	0.175 (0.03; 0.32)	0.019	0.164 (0.02; 0.31)	0.030	0.097 (−0.06; 0.25)	ns
	HbA1c in 3.trimester	0.214 (0.08; 0.35)	0.002	0.202 (0.06; 0.34)	0.006	0.162 (0.01; 0.31)	0.037
	HbA1c partum	0.269 (0.13; 0.41)	< 0.001	0.217 (0.08; 0.35)	0.002	0.196 (0.05; 0.34)	0.008
Cord-blood insulin							
	FG at oGTT	0.114 (−0.04; 0.27)	ns	0.018 (−0.14; 0.18)	ns	0.012 (−0.15; 0.18)	ns
	1-h glucose at oGTT	0.039 (−0.12; 0.20)	ns	−0.027 (− 0.18; 0.13)	ns	0.003 (− 0.15; 0.16)	ns
	2-h glucose at oGTT	0.047 (−0.11; 0.20)	ns	0.037 (−0.11; 0.19)	ns	0.065 (−0.09; 0.22)	ns
	AUC glucose at oGTT	0.041 (−0.12; 0.20)	ns	−0.012 (− 0.17; 0.14)	ns	0.026 (− 0.14; 0.19)	ns
	Mean FG at 32nd GW	0.241 (0.08; 0.40)	0.004	0.243 (0.07; 0.41)	0.005	0.231 (0.06; 0.41)	0.010
	Mean PPG at 32nd GW	0.225 (0.07; 0.38)	0.005	0.116 (−0.04; 0.28)	ns	0.085 (−0.08; 0.25)	ns
	Mean FG at 36th GW	0.224 (0.07; 0.38)	0.006	0.194 (0.04; 0.35)	0.012	0.172 (0.01; 0.33)	0.035
	Mean PPG at 36th GW	0.143 (−0.02; 0.31)	ns	0.080 (−0.07; 0.24)	ns	0.037 (−0.12; 0.20)	ns
	HbA1c in 3.trimester	0.315 (0.16; 0.48)	< 0.001	0.209 (0.04; 0.37)	0.014	0.180 (−0.01; 0.37)	ns
	HbA1c partum	0.386 (0.24; 0.54)	< 0.001	0.300 (0.15; 0.45)	< 0.001	0.282 (0.11; 0.45)	0.001
Cord-blood leptin							
	FG at oGTT	0.157 (0.01; 0.31)	0.042	0.065 (−0.09; 0.22)	ns	0.034 (− 0.13; 0.20)	ns
	1-h glucose at oGTT	0.084 (−0.07; 0.24)	ns	0.054 (−0.10; 0.21)	ns	0.072 (−0.09; 0.23)	ns
	2-h glucose at oGTT	0.134 (−0.02; 0.29)	ns	0.154 (0.01; 0.30)	ns	0.169 (0.02; 0.32)	0.028
	AUC glucose at oGTT	0.124 (−0.03; 0.28)	ns	0.107 (−0.05; 0.26)	ns	0.122 (−0.04; 0.28)	ns
	Mean FG at 32nd GW	0.204 (0.05; 0.36)	0.012	0.172 (0.01; 0.34)	0.043	0.149 (−0.02; 0.32)	ns
	Mean PPG at 32nd GW	0.194 (0.04; 0.35)	0.015	0.131 (−0.03; 0.29)	ns	0.122 (−0.04; 0.28)	ns
	Mean FG at 36th GW	0.188 (0.03; 0.35)	0.020	0.122 (−0.04; 0.28)	ns	0.073 (−0.10; 0.24)	ns
	Mean PPG at 36th GW	0.104 (−0.06; 0.27)	ns	0.030 (−0.13; 0.19)	ns	0.004 (−0.16; 0.17)	ns
	HbA1c in 3.trimester	0.290 (0.14; 0.44)	< 0.001	0.190 (0.03; 0.35)	0.021	0.188 (0.01; 0.37)	0.041
	HbA1c partum	0.350 (0.20; 0.50)	< 0.001	0.261 (0.11; 0.42)	0.001	0.259 (0.09; 0.43)	0.003

Abbreviations: *FG* Fasting glucose, *oGTT* Oral glucose tolerance test, *ns* Non-significant, *AUC* Area under the curve, *GW* Gestational week, *PPG* Postprandial glucose, *HbA1c* Glycated hemoglobin A1c

^a^Model I: Adjusted for maternal age, socio-economic status, ethnic origin, smoking during pregnancy, parity, height, type of therapy, gestational age, mode of delivery and infant’s sex.

Model II: Model I adjustment plus prepregnancy body weight and net gestational weight gain

### Identification of major categorical parental determinants of anthropometric birth and cord-blood hormone outcomes

Forward step-wise regression analyses with key paternal and maternal general, anthropometric, and metabolic parameters in (clinically) relevant categories were then performed to identify major predictors for critical birth outcomes (Table 5). Aside from classical birth weight determining factors, e.g. term delivery, smoking during pregnancy etc., maternal, but not paternal, anthropometric and metabolic variables, respectively, appeared as predictors. However, maternal anthropometry, i.e., prepregnant overweight/obesity and excessive GWG (according to IOM criteria), was positively related only to relative birth weight and explained around 2–3% of the variance (Table 5). On the contrary, maternal glucose during 3rd trimester showed a variety of associations. For example, mean FG at 32nd weeks’ gestation > 90 mg/dL was the strongest factor for placental weight (6% of variance) and a predictor for birth weight and cord-blood insulin (Table 5). A FG value > 90 mg/dL at oGTT was a significant positive factor for cord-blood leptin at birth (3% of variance). Unexpectedly, 1-h glucose value at oGTT above the guideline cut-off (> 180 mg/dL) was associated with lower instead of higher birth weight and relative birth weight, explaining 2–4% of variances. As shown in Table 5, HbA1c at delivery was a particular strong predictor for higher cord-blood insulin and leptin levels (7% of variance).

**Table 5:** Maternal categorical predictor variables affecting anthropometric birth outcomes and cord-blood hormones

Outcome variable	Predictor variable^a^	B (95% CI)	*P*-value	Adj. Sr^2^
Placental weight	Mean FG at 32nd GW > 90 mg/dL	0.487 (0.18; 0.80)	0.002	0.057
	Term delivery	0.934 (0.30; 1.57)	0.004	0.040
	Cesarean section	0.424 (0.11; 0.74)	0.008	0.036
Birth weight	Term delivery	1.400 (0.80; 2.00)	< 0.001	0.118
	1-h glucose at oGTT > 180 mg/dL	−0.501 (−0.86; −0.15)	0.006	0.035
	Smoking in pregnancy	−0.561 (− 0.96; − 0.17)	0.006	0.029
	Male sex	0.333 (0.05; 0.62)	0.022	0.023
	Mean FG at 32nd GW >90 mg/dL	0.297 (0.01; 0.59)	0.044	0.017
Relative birth weight	Term delivery	1.457 (0.86; 2.06)	< 0.001	0.121
	Excessive GWG	0.332 (0.04; 0.62)	0.026	0.029
	Smoking in pregnancy	−0.565 (−0.97; −0.16)	0.007	0.025
	Prepregnancy BMI > 25 kg/m^2^	0.319 (0.02; 0.61)	0.034	0.024
	1-h glucose at oGTT > 180 mg/dL	−0.375 (− 0.74; − 0.01)	0.042	0.016
	Male sex	0.295 (0.01; 0.58)	0.044	0.016
Cord-blood insulin	Cesarean section	0.679 (0.38; 0.97)	< 0.001	0.170
	HbA1c partum > 6%	0.460 (0.09; 0.83)	0.016	0.066
	Non-European ethnicity	0.438 (0.13; 0.75)	0.006	0.043
	Mean FG at 32nd GW >90 mg/dL	0.321 (0.02; 0.63)	0.038	0.020
Cord-blood leptin	HbA1c partum > 6%	0.470 (0.08; 0.86)	0.017	0.070
	Non-European ethnicity	0.519 (0.19; 0.85)	0.002	0.048
	Male sex	−0.395 (−0.71; −0.08)	0.013	0.042
	FG at oGTT >90 mg/dL	0.339 (0.03; 0.65)	0.034	0.025

Unstandardized B coefficients with 95% confidence interval and adjusted semipartial correlation coefficients (Sr^2^), which estimate the contribution of each factor to the variance of the outcome variable, are shown

Abbreviations: *FG* Fasting glucose, *GW* Gestational week, *oGTT* Oral glucose tolerance test, *GWG* Gestational weight gain, *BMI* Body-mass-index, *HbA1c* Glycated hemoglobin A1c

^a^Included categorical variables: Paternal SES (lower/higher), maternal SES (lower/higher), parity (nulliparous/multiparous), ethnicity (European/Non-European), smoking in pregnancy (no/yes), type of therapy (diet/insulin), mode of delivery (Cesarean section no/yes), gestational age (preterm/term), infant’s sex (female/male), paternal BMI > 25 kg/m^2^ (no/yes), paternal BMI > 30 kg/m^2^ (no/yes), maternal prepregnancy BMI > 25 kg/m^2^ (no/yes), prepregnancy BMI > 30 kg/m^2^ (no/yes), inadequate GWG (no/yes), excessive GWG (no/yes), FG at oGTT > 90 mg/dL (no/yes), 1-h glucose at oGTT > 180 mg/dL (no/yes), 2-h glucose at oGTT > 155 mg/dL (no/yes), mean FG at 32nd weeks’ gestation > 90 mg/dL (no/yes), mean FG at 36th weeks’ gestation > 90 mg/dL (no/yes), HbA1c in third trimester > 6% (no/yes) and partum > 6% (no/yes).

Finally, logistic regression analysis was performed. Because of low numbers of adverse clinical endpoints, analysis was restricted to the outcome LGA. Indeed, crude, unadjusted evaluation revealed increased prepregnancy BMI as risk factor for LGA (OR: 1.53, 95% CI: 1.06–2.20, *P* = 0.023). However, after adjustment for 3rd trimester glucose according to Model II variables (see above), neither pregravid BMI (OR: 1.39, 95% CI: 0.80–2.40) nor total GWG (OR: 1.52, 95% CI: 0.95–2.42) nor net GWG (OR: 1.34, 95% CI: 0.83–2.15) were significantly related to LGA.

**Fig. 3:**
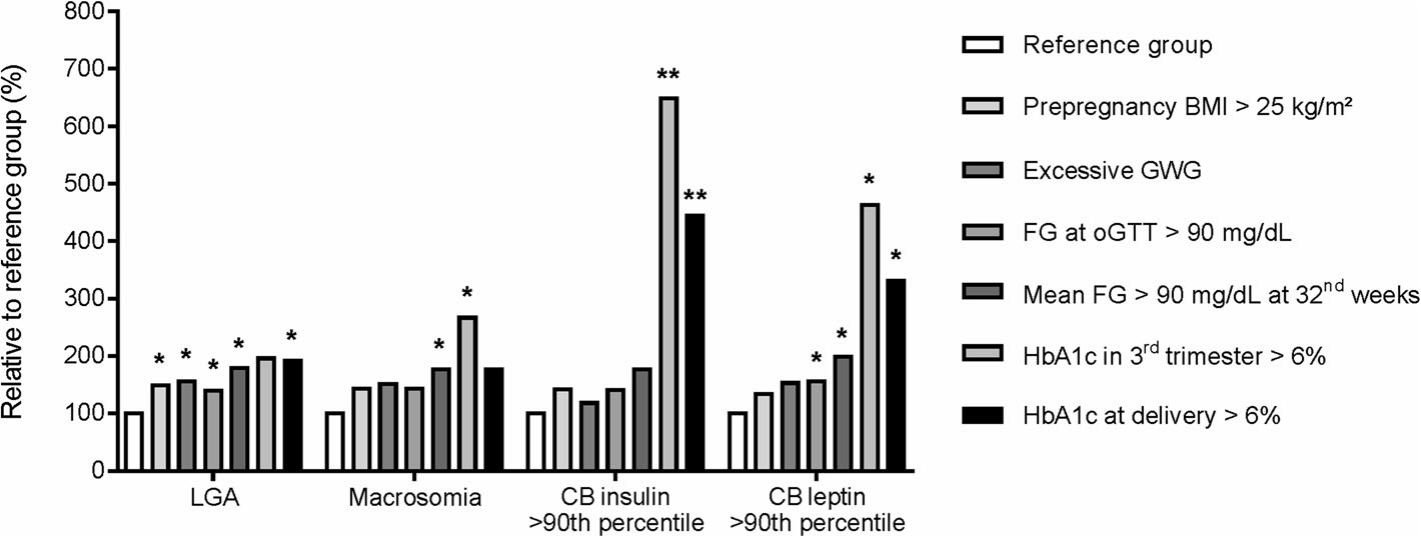
Relative percentages of significant maternal anthropometric and metabolic variables/exposures in newborns with unfavorable outcomes. Frequencies of maternal key anthropometric and metabolic variables/exposures in newborns with unfavorable outcomes relative to respective reference groups (LGA/Macrosomia: Reference group consists of appropriate-for-gestational age and non-macrosomic infants; CB insulin/leptin: Reference group consists of all cases <90th percentiles) are shown. Significant differences are indicated as * *P* < 0.05, ** *P* < 0.001. Abbreviations: LGA: Large-for-gestational age, CB: Cord-blood, BMI: Body-mass-Index, GWG: Gestational weight gain, FG: Fasting glucose, oGTT: Oral glucose tolerance test, HbA1c: Glycated hemoglobin A1c

Additionally, to further illustrate observations for clinically unfavorable neonatal anthropometric (LGA and macrosomia) and cord-blood outcomes (insulin and leptin > 90th percentiles), percentages of relevant anthropometric and metabolic variables/exposures, grouped according to clinical/guideline cut-offs, were compared to respective internal reference groups which did not show these adverse outcomes (Fig. 3). Following this approach, maternal prepregnancy overweight/obesity and excessive GWG only showed significantly higher relative frequencies regarding LGA (Fig. 3). In contrast, parameters of maternal hyperglycemia during 3rd trimester were associated with all unfavorable endpoints. Percentages of 3rd trimester glucose variables above cut-offs, in particular mean FG at 32nd weeks’ gestation > 90 mg/dL and HbA1c > 6% in 3rd trimester as well as at delivery, were significantly higher in offspring with LGA and macrosomia, respectively. Notably, women with macrosomic offspring exceeded 2.5-times more often the cut-off for HbA1c in 3rd trimester (Fig. 3). Furthermore, both cord-blood insulin as well as leptin > 90th percentile showed strong positive associations with maternal 3rd trimester hyperglycemia, but neither with pregravid BMI nor excessive GWG. Percentages above the cut-offs of FG at oGTT and mean FG at 32nd weeks’ gestation (> 90 mg/dL for both) were significantly higher in offspring with cord-blood leptin > 90th percentile. Finally, relative frequencies above the cut-off for HbA1c at both investigated time points were even increased up to 3- to 6-fold in offspring with cord-blood insulin and leptin > 90th percentile, respectively (Fig. 3).

## Discussion

Maternal overweight/obesity and GDM are associated with adverse short- and long-term outcomes in the offspring [5, 7], the latter identified to be related especially to increased birth weight, insulin and leptin. In this prospective observational study of women with treated GDM the influence of maternal anthropometry on respective birth outcomes was strongly dependent on maternal glycemia in late pregnancy. After adjustment to 3rd trimester glucose metabolism, no significant effect of maternal anthropometry was observed on placental weight, birth weight, relative birth weight (weight-to-length ratio), and cord-blood insulin and leptin, respectively. On the contrary, 3rd trimester glucose variables, and only marginally FG at oGTT screening, showed clear positive associations with these outcomes, even after adjustment for a variety of general as well as anthropometric parental measures. Thus, maternal (hyper-) glycemia in 3rd trimester appears to be the key target for managing and avoiding alterations of fetal growth (adiposity) and hormone levels affecting long-term health.

Our observations are in line with data from other cohort studies showing that treatment of GDM, i.e., achieving good metabolic control, can significantly reduce the potential effects of maternal (prepregnancy) BMI status on adverse anthropometric perinatal outcomes [37–39]. Beyond their focus on LGA and macrosomia, the present study additionally highlights the influence of maternal 3rd trimester glycemia on a wide birth weight and relative birth weight range. Considering, in general, a linear increase of later overweight risk with increasing birth weight [11], strict management of maternal glycemia throughout the fetal growth spectrum may therefore be beneficial for the offspring, to protect their genuine growth potential. Other studies concluded that maternal obesity per se is a major factor or even better predictor than maternal glycemia to manage fetal (over-) growth in women with treated GDM [40–42]. Similar to the present cohort, obesity was accompanied in two of these studies by higher glucose levels compared to normal weight individuals [40, 41]. However, only group comparisons [41] and no adjustments for general covariates and/or maternal glycemia in later pregnancy were performed [40, 41]. Thus, the relative contributions of maternal weight status vs. glucose may have been different after all, and might have depend lastly on the degree of 3rd trimester glycemia in these cohorts, too. Similarly, in another retrospective study [42] critical information about the relationship between maternal BMI and glucose was not provided and HbA1c levels in 3rd trimester and at delivery were not available. Interestingly, however, although maternal obesity was clearly associated with fetal overgrowth, it was concluded that during the period of maximum in utero growth, i.e., 3rd trimester, maternal glycemia was the predominant factor.

Furthermore, some studies reported that increased maternal GWG is an independent factor for adverse anthropometric outcomes in women with GDM [43–46]. Again, however, maternal glycemia in later pregnancy or even only at GDM screening was not considered in the final analyses, with the exception of one study [43]. Interestingly enough, here multivariable analysis revealed that total maternal weight gain during pregnancy was finally only of minor significance while increased mean blood glucose during the treatment period was associated with a 2-fold risk for LGA outcome. Hence, the effects of maternal GWG seem to decisively depend on maternal glycemia, as observed in the present study. Total and 3rd trimester GWG, both comprising fetal weight, were initially associated with birth weight and relative birth weight but no longer after adjustment to 3rd trimester glucose variables. Moreover, genuine maternal weight gain (net GWG) per se showed no relation to any outcome. Based on these observations, future studies should consider adjustment to maternal glycemia but also net GWG for clear-cut analyses and interpretation of the impact of maternal anthropometry for the outcome.

In addition to neonatal anthropometry, our study also shows that maternal glycemia in 3rd trimester, but not maternal anthropometry per se, is independently related to critical cord-blood hormones shown to promote fetal ‘malprogramming’. While causal factors for adverse neonatal anthropometric outcomes are intensively studied, determinants of cord-blood insulin and leptin are less considered in clinical studies, although probably even more important mechanistically for the long-term development of metabolic disorders [8, 14, 20, 47–49]. Especially, hyperinsulinism has been shown to be crucial for long-term ‘malprogramming’ of the child towards ‘diabesity’ predisposition [20, 47, 48]. Furthermore, cord-blood leptin correlates with neonatal adiposity [50], and might act as a growth factor in utero itself [51]. There is strong evidence that maternal obesity and diabetes are associated with higher cord-blood insulin and leptin concentrations [52–55]. With regard to maternal glycemia it has been shown recently that both hormones are related to fasting and post load glucose at oGTT, adjusted for maternal BMI, in a multiethnic cohort with and without GDM [56]. Consistently, the present study indicates a strong influence of maternal glucose, but not BMI or GWG per se*,* on both hormones. Here, however, not glucose at the time of oGTT but in later pregnancy appeared critically linked to adverse birth outcomes. Since both clinical as well as experimental studies demonstrated the particular importance of, especially, hyperinsulinism for long-term ‘diabesity programming’, this observation seems to deserve particular attention [14, 20, 57].

The HAPO study clearly showed a linear positive relationship between maternal glucose concentrations at oGTT screening, in particular FG, and critical birth outcomes, e.g. birth weight and cord-blood C-peptide > 90th percentiles, respectively [16]. Moreover, it is suggested from HAPO that maternal obesity and GDM are independent risk factors for these outcomes, while the odds are additive and higher with increasing glycemia [15]. First, this implies that maternal glycemia is generally a major factor for fetal (over-) growth and hyperinsulinemia and further that a strong interaction exists between maternal BMI and glucose metabolism, while the relative contributions are difficult to tease apart. In HAPO, and a number of related studies addressing the influence of maternal anthropometry on neonatal outcomes, adjustment was made, however, only to oGTT glucose data [15, 58, 59], if performed at all [60, 61], while consideration of later maternal glycemia appears critical for analyses and interpretation. As indicated by the present study, the effects of maternal BMI and GWG, respectively, depend strongly on glucose metabolism not at oGTT but in later pregnancy, contributing significantly to fetal (over-) growth and critical cord-blood outcomes, respectively. Therefore, considering increasing insulin resistance in later pregnancy, regular glucose measurements in overweight/obese women appear to be recommendable even after a negative oGTT screening test. To date, however, research studies considering maternal glycemia in the 3rd trimester are very rare.

Although our data might appear to challenge the impact of maternal overweight for adverse birth outcomes, this would not be correct. Of course, maternal overweight/obesity and increased gestational weight gain play an important detrimental role, just due to tight links with glucose metabolism (insulin sensitivity). As shown here, not all initial associations of adverse birth outcomes with maternal 3rd trimester glucose withstood adjustment to maternal anthropometry. Furthermore, applying step-wise regression analyses with common categorical parameters to our dataset, pregravid overweight/obesity and excessive GWG remained predictors for relative birth weight and showed higher percentages in LGA offspring as compared to the internal reference group. However, all of these effects were rather weak and could not be confirmed using continuous variable analyses. Compared to maternal anthropometric parameters, glucose in 3rd trimester was clearly better related to birth outcomes, in particular placental weight, macrosomia and cord-blood insulin as well as leptin, the latter mainly reflecting neonatal adiposity. Taken together, our data therefore indicates that the impact of (higher) maternal weight status and GWG on critical birth outcomes is ultimately to a major extent attributable to maternal glycemia in late pregnancy, but not at the time of oGTT, i.e., potentially even independent of a negative oGTT screening test. This might have far ranging implications for clinical care.

Our study suggests, that especially maternal glucose at 32nd weeks’ gestation as well as HbA1c levels, integrative characterizing 3rd trimester glycemia, are positively related to fetal (over-) growth and (adverse) cord-blood hormone outcomes, respectively. HbA1c at delivery showed particular pronounced effects. This appears plausible, as in general the 3rd trimester is the period of major fetal growth, e.g. due to accelerated fat accumulation, and intrauterine differentiation and maturation [19]. Furthermore, these observations indicate that cumulative maternal hyperglycemia during late pregnancy, characterized by HbA1c, plays a crucial role, which is in agreement with other studies [62–64]. Ensenauer and colleagues [63] reported that even non-GDM obese women with elevated HbA1c at delivery showed increased odds for LGA newborns and cord-blood C-peptide, also after adjustment for maternal prepregnancy BMI and GWG. Interestingly, in the present study, major relations between maternal glycemia and placental weight were observed especially for glucose at 32nd week’ gestation. Because placental weight strongly determines/correlates with fetal weight, as shown here and in other reports [36, 65], glucose-dependent placental growth may be an important pathway by which maternal 3rd trimester glycemia influences fetal growth.

Unexpectedly, glucose values at oGTT were, with few exceptions, not significantly related to birth outcomes, which may be a consequence of GDM treatment. Even more surprising was the negative association between 1-h glucose at oGTT above the guideline cut-off (> 180 mg/dL) and birth weight/relative birth weight in step-wise regression analyses on categorical variables. While the majority of women in the total cohort exceeded this threshold, earlier and stricter treatment was applied to these women, possibly resulting in relatively lower birth weights. Moreover, relative frequencies of multiparous gestation, insulin therapy, preterm delivery and SGA outcome were non-significantly higher in women with glucose > 180 mg/dL at oGTT as compared to those below this cut-off. Possibly, all of this might cumulatively provide an explanation for this finding, which was not observed in the other analyses here.

Also worthy to note, paternal anthropometry has been suggested to be directly related to fetal growth and birth weight [66]. As no associations were found in the present study, no support can be given to this hypothesis, in line with other observations speaking clearly against relevant paternal anthropometric determination of birth weight [67]. Interestingly, however, our data indicate “assortative mating” regarding BMI and SES. This may also appear in other studies and, thus, proper adjustment to respective paternal factors should be performed.

Lastly, like all observational studies also the present one had some limitations, which may have influenced the results. First, as only women with sufficient German/English language skills were included into the study, a selection bias could be present here, which appears unavoidable. Secondly, maternal self-reported weight before pregnancy was used for data evaluation. General concordance, however, has been shown for self-reported vs. measured maternal prepregnancy weight [68]. Furthermore, GWG calculations were based on pregravid weight and the last gestational weight measured within one week prior to delivery, which is not totally accurate but common practice in such studies. In addition, due to the design of the study, only placental weight and no other placental characteristics, e.g. shape, size etc., were assessed.

Of note, in BMI group comparisons, the anthropometric outcomes birth weight, relative birth weight and LGA were similar in prepregnancy overweight and obese mothers and did not further increase in the latter, despite higher glycemia in 3rd trimester in the obese. Multiple reasons may explain these observations. For example, factors generally associated with fetal growth restriction and lower birth weight were (much) more abundant in the obese group, e.g. smoking, pregnancy-related hypertensive disorders, preterm delivery, and the mean gestational age at delivery was lower. As highlighted above, adjustment to such potential confounder variables is crucial for analyses and interpretation. Therefore, among a number of covariates, all general predictor variables, e.g. those identified in step-wise regression, were included in the adjustment models. Finally, since sex-specific differences were observed regarding determinants of the investigated fetal/birth outcomes [36], adjustment was made to infant’s sex. Interestingly, offspring’s gender, indeed, appeared as potential co-predictor for birth weight and cord-blood leptin, with males more predisposed to higher weight but, remarkably, lower leptin levels at birth. Similar was already observed [54, 69] and should be further explored in future studies.

## Conclusions

In summary and conclusion, in a cohort of women with treated GDM maternal BMI and GWG were not associated with birth outcomes critical for ‘perinatal programming’ after consideration of maternal 3rd trimester glucose metabolism. While glucose at oGTT showed almost no relationships with endpoints, glucose metabolism in later pregnancy was strongly and independently associated with anthropometric and cord-blood hormone outcomes at birth. Thus, maternal overweight does not independently contribute to the detrimental birth outcomes addressed here, while it appears mandatory to strictly manage and scientifically consider, respectively, 3rd trimester glucose in gestational care and future research studies on the short- and long-term consequences of GDM and/or overweight/obesity during pregnancy.

## Abbreviations

AGA: Appropriate-for-gestational age
AUC: Area under the curve
BMI: Body-mass-index
CB: Cord-blood
CI: Confidence interval
CS: Cesarean section
EaCH: Early CHARITÉ study
FG: Fasting glucose
GDM: Gestational diabetes mellitus
GW: Gestational week
GWG: Gestational weight gain
HAPO: Hyperglycemia and adverse pregnancy outcome study
HbA1c: Glycated hemoglobin A1c
IOM: Institute of Medicine
LGA: Large-for-gestational age
Ns: Non-significant
oGTT: oral Glucose tolerance test
OR: Odds ratio
PPG: Postprandial glucose
SD: Standard deviation
SES: Socio-economic status
SGA: Small-for-gestational age
WHO: World Health Organization

## References

[1] O’Sullivan EP, Avalos G, O’Reilly M, Dennedy MC, Gaffney G, Dunne F (2011). Atlantic diabetes in pregnancy (DIP): the prevalence and outcomes of gestational diabetes mellitus using new diagnostic criteria. Diabetologia.

[2] Sacks DA, Hadden DR, Maresh M, Deerochanawong C, Dyer AR, Metzger BE (2012). Frequency of gestational diabetes mellitus at collaborating centers based on IADPSG consensus panel-recommended criteria: the hyperglycemia and adverse pregnancy outcome (HAPO) study. Diabetes Care.

[3] Melchior H, Kurch-Bek D, Mund M (2017). Population-based analysis of a nationwide screening program. Dtsch Arztebl Int.

[4] Flegal KM, Carroll MD, Odgen CL, Curtin LR (2010). Prevalence and trends in obesity among US adults, 1999-2008. JAMA.

[5] Catalano PM, Ehrenberg HM (2006). The short- and long-term implications of maternal obesity on the mother and her offspring. BJOG.

[6] Metzger BE, Buchanan TA, Coustan DR, De Leiva A, Dunger DB, Hadden DR (2007). Summary and recommendations of the fifth international workshop-conference on gestational diabetes mellitus. Diabetes Care.

[7] Reece EA (2010). The fetal and maternal consequences of gestational diabetes mellitus. J Matern Fetal Neonatal Med.

[8] Plagemann A (2011). Maternal diabetes and perinatal programming. Early Hum Dev.

[9] Barker DJP (2007). Obesity and early life. Obes Rev.

[10] Harder T, Rodekamp E, Schellong K, Dudenhausen JW, Plagemann A (2007). Birth weight and subsequent risk of type 2 diabetes: a meta-analysis. Am J Epidemiol.

[11] Schellong K, Schulz S, Harder T, Plagemann A (2012). Birth weight and long-term overweight risk: systematic review and a meta-analysis including 643,902 persons from 66 studies and 26 countries globally. PLoS One.

[12] Van Assche FA, Holemans K, Aerts L (2001). Long-term consequences for offspring of diabetes during pregnancy. Br Med Bull.

[13] Fetita LS, Sobngwi E, Serradas P, Calvo F, Gautier JF (2006). Consequences of fetal exposure to maternal diabetes in offspring. J Clin Endocrinol Metab.

[14] Plagemann A (2008). A matter of insulin: developmental programming of body weight regulation. J Matern Fetal Neonatal Med.

[15] Catalano PM, McIntyre HD, Cruickshank JK, McCance DR, Dyer AR, Metzger BE (2012). The hyperglycemia and adverse pregnancy outcome study: associations of GDM and obesity with pregnancy outcomes. Diabetes Care.

[16] HAPO Study Cooperative Research Group (2008). Hyperglycemia and adverse pregnancy outcomes. N Engl J Med.

[17] Crowther CA, Hiller JE, Moss JR, McPhee AJ, Jeffries WS, Robinson JS (2005). Effect of treatment of gestational diabetes mellitus on pregnancy outcomes. N Engl J Med.

[18] Landon MB, Spong CY, Thom E, Carpenter MW, Ramin SM, Casey B (2009). A multicenter, randomized trial of treatment for mild gestational diabetes. N Engl J Med.

[19] Moore KL, Persaud TV, Torchia MG (2011). The developing human.

[20] Silverman BL, Metzger BE, Cho NH, Loeb CA (1995). Impaired glucose tolerance in adolescent offspring of diabetic mothers: relationship to fetal hyperinsulinism. Diabetes Care.

[21] Clausen TD, Mathiesen ER, Hansen T, Pedersen O, Jensen DM, Lauenborg J (2009). Overweight and the metabolic syndrome in adult offspring of women with diet-treated gestational diabetes mellitus or type 1 diabetes. J Clin Endocrinol Metab.

[22] World MA (2004). World medical association declaration of Helsinki: ethical principles for medical research involving human subjects. J Int Bioethique.

[23] Rodekamp E, Harder T, Kohlhoff R, Franke K, Dudenhausen JW, Plagemann A (2005). Long-term impact of breast-feeding on body weight and glucose tolerance in children of diabetic mothers: role of the late neonatal period and early infancy. Diabetes Care.

[24] Plagemann A, Harder T, Rodekamp E, Kohlhoff R (2012). Rapid neonatal weight gain increases risk of childhood overweight in offspring of diabetic mothers. J Perinat Med.

[25] Gemeinsamer Bundesausschuss. Mutterpass. https://www.g-ba.de/downloads/17-98-4071/2016-02-16_Mutterpass_englisch_WEB_WZ.pdf. Accessed 12 July 2017.

[26] Rasmussen KM, Yaktine AL (2009). Weight gain during pregnancy: reexamining the guidelines.

[27] Deutsche Gesellschaft für Gynäkologie und Geburtshilfe. Diabetes und Schwangerschaft. https://www.dggg.de/fileadmin/documents/leitlinien/archiviert/beteiligt/057023_Diabetes_und_Schwangerschaft/057023_2008.pdf. Accessed 15 July 2017.

[28] Carpenter MW, Coustan DR (1982). Criteria for screening tests for gestational diabetes. Am J Obstet Gynecol.

[29] American Diabetes Association. Standards of medical care in diabetes - 2016. Section 12. Management of diabetes in pregnancy. Diabetes Care 2016;39 Suppl 1:S94–S98. 10.2337/dc16-S015 26696688

[30] Deutsche Gesellschaft für Gynäkologie und Geburtshilfe. Diagnostik und Therapie hypertensiver Schwangerschaftserkrankungen. https://www.dggg.de/fileadmin/documents/leitlinien/archiviert/federfuehrend/015018_Diagnostik_und_Therapie_hypertensiver_Schwangerschaftserkrankungen/015018_2008.pdf. Accessed 15 July 2017.

[31] HAPO Study Cooperative Research Group (2002). The hyperglycemia and adverse pregnancy outcome (HAPO) study. Int J Gynaecol Obstet.

[32] Alshimmiri MM, Hammoud MS, Al-Saleh EA, Alsaeid KMS (2003). Ethnic variations in birthweight percentiles in Kuwait. Paediatr Perinat Epidemiol.

[33] Voigt M, Fusch C, Olbertz D, Hartmann K, Rochow N, Renken C (2006). Analyse des Neugeborenenkollektivs der Bundesrepublik Deutschland. Geburtshilfe Frauenheilkd.

[34] Kurtoǧlu S, Hatipoǧlu N, Mazicioǧlu MM, Akin MA, Çoban D, Gökoǧlu S (2012). Body weight, length and head circumference at birth in a cohort of Turkish newborns. J Clin Res Pediatr Endocrinol.

[35] Templeton GF (2011). A two-step approach for transforming continuous variables to normal: implications and recommendations for IS research. CAIS.

[36] O ‘Tierney-Ginn P, Presley L, Minium J, Hauguel-de Mouzon S, Catalano PM (2014). Sex-specific effects of maternal anthropometrics on body composition at birth. Am J Obstet Gynecol.

[37] Langer O, Yogev Y, Xenakis EMJ, Brustman L (2005). Overweight and obese in gestational diabetes: the impact on pregnancy outcome. Am J Obstet Gynecol.

[38] Yogev Y, Langer O (2008). Pregnancy outcome in obese and morbidly obese gestational diabetic women. Eur J Obstet Gynecol Reprod Biol.

[39] Casey BM, Mele L, Landon MB, Spong CY, Ramin SM, Wapner RJ (2015). Does maternal body mass index influence treatment effect in women with mild gestational diabetes?. Am J Perinatol.

[40] Roman AS, Rebarber A, Fox NS, Klauser CK, Istwan N, Rhea D (2011). The effect of maternal obesity on pregnancy outcomes in women with gestational diabetes. J Matern Fetal Neonatal Med.

[41] Schaefer-Graf UM, Heuer R, Kilavuz O, Pandura A, Henrich W, Vetter K (2002). Maternal obesity not maternal glucose values correlates best with high rates of fetal macrosomia in pregnancies complicated by gestational diabetes. J Perinat Med.

[42] Schaefer-Graf UM, Kjos SL, Kilavuz O, Plagemann A, Brauer M, Dudenhausen JW (2003). Determinants of fetal growth at different periods of pregnancies complicated by gestational diabetes mellitus or impaired glucose tolerance. Diabetes Care.

[43] Most O, Langer O (2012). Gestational diabetes: maternal weight gain in relation to fetal growth, treatment modality, BMI and glycemic control. J Matern Fetal Neonatal Med.

[44] Berggren EK, Stuebe AM, Boggess KA (2015). Excess maternal weight gain and large for gestational age risk among women with gestational diabetes. Am J Perinatol.

[45] Egan AM, Dennedy MC, Al-Ramli W, Heerey A, Avalos G, Dunne F (2014). ATLANTIC-DIP: excessive gestational weight gain and pregnancy outcomes in women with gestational or pregestational diabetes mellitus. J Clin Endocrinol Metab.

[46] Miao M, Dai M, Zhang Y, Sun F, Guo X, Sun G (2017). Influence of maternal overweight, obesity and gestational weight gain on the perinatal outcomes in women with gestational diabetes mellitus. Sci Rep.

[47] Plagemann A, Harder T, Kohlhoff R, Rohde W, Dörner G (1997). Overweight and obesity in infants of mothers with long-term insulin-dependent diabetes or gestational diabetes overweight and obesity in infants of mothers with long-term insulin-dependent diabetes or gestational diabetes. Int J Obes Relat Metab Disord.

[48] Plagemann A, Harder T, Kohlhoff R, Rohde W, Dörner G (1997). Glucose tolerance and insulin secretion in children of mothers with pregestational IDDM or gestational diabetes. Diabetologia.

[49] Freinkel N, Metzger BE (1978). Pregnancy as a tissue culture experience: the critical implications of maternal metabolism for fetal development; in pregnancy metabolism, diabetes and the fetus. Ciba Found Symp.

[50] Donnelly JM, Lindsay KL, Walsh JM, Horan M, Molloy EJ, McAuliffe FM (2015). Fetal metabolic influences of neonatal anthropometry and adiposity. BMC Pediatr.

[51] Christou H, Connors JM, Ziotopoulou M, Hatzidakis V, Papathanassoglou E, Ringer SA (2001). Cord blood leptin and insulin-like growth factor levels are independent predictors of fetal growth. J Clin Endocrinol Metab.

[52] Shekhawat PS, Garland JS, Shivpuri C, Mick GJ, Sasidharan P, Pelz CJ (1998). Neonatal cord blood leptin: its relationship to birth weight, body mass index, maternal diabetes, and steroids. Pediatr Res.

[53] Tapanainen P, Leinonen E, Ruokonen A, Knip M (2001). Leptin concentrations are elevated in newborn infants of diabetic mothers. Horm Res.

[54] Okereke NC, Uvena-Celebrezze J, Hutson-Presley L, Amini SB, Catalano PM (2002). The effect of gender and gestational diabetes mellitus on cord leptin concentration. Am J Obstet Gynecol.

[55] Catalano PM, Presley L, Minium J, Hauguel-de Mouzon S (2009). Fetuses of obese mothers develop insulin resistance in utero. Diabetes Care.

[56] Lawlor DA, West J, Fairley L, Nelson SM, Bhopal RS, Tuffnell D (2014). Pregnancy glycaemia and cord-blood levels of insulin and leptin in Pakistani and white British mother-offspring pairs : findings from a prospective pregnancy cohort. Diabetologia.

[57] Weiss PMA, Scholz HS, Haas J, Tamussino KE, Seissler J, Borkenstein MH (2000). Long-term follow-up of infants of mothers with type 1 diabetes. Diabetes Care.

[58] Ong KK, Diderholm B, Salzano G, Wingate D, Hughes IA, MacDougall J (2008). Pregnancy insulin, glucose, and BMI contribute to birth outcomes in nondiabetic mothers. Diabetes Care.

[59] Badon SE, Dyer AR, Josefson JL (2014). Gestational weight gain and neonatal adiposity in the hyperglycemia and adverse pregnancy outcome study-north American region. Obesity (Silver Spring).

[60] Ehrenberg HM, Mercer BM, Catalano PM (2004). The influence of obesity and diabetes on the prevalence of macrosomia. Am J Obstet Gynecol.

[61] Black MH, Sacks DA, Xiang AH, Lawrence JM (2013). The relative contribution of prepregnancy overweight and obesity, gestational weight gain, and IADPSG-defined gestational diabetes mellitus to fetal overgrowth. Diabetes Care.

[62] Damm P, Mersebach H, Råstam J, Kaaja R, Hod M, McCance DR (2014). Poor pregnancy outcome in women with type 1 diabetes is predicted by elevated HbA1c and spikes of high glucose values in the third trimester. J Matern Fetal Neonatal Med.

[63] Ensenauer R, Brandlhuber L, Burgmann M, Sobotzki C, Zwafink C, Anzill S (2015). Obese nondiabetic pregnancies and high maternal glycated hemoglobin at delivery as an indicator of offspring and maternal postpartum risks : the prospective PEACHES mother-child cohort. Clin Chem.

[64] Cyganek K, Skupien J, Katra B, Hebda-Szydlo A, Janas I, Trznadel-Morawska I (2017). Risk of macrosomia remains glucose-dependent in a cohort of women with pregestational type 1 diabetes and good glycemic control. Endocrine.

[65] Ouyang F, Parker MG, Luo ZC, Wang X, Zhang HJ, Jiang F (2016). Maternal BMI, gestational diabetes, and weight gain in relation to childhood obesity: the mediation effect of placental weight. Obesity (Silver Spring).

[66] Griffiths LJ, Dezateux C, Cole TJ (2007). Differential parental weight and height contributions to offspring birthweight and weight gain in infancy. Int J Epidemiol.

[67] Brooks AA, Johnson MR, Steer PJ, Pawson ME, Abdalla HI (1995). Birth weight: nature or nurture?. Early Hum Dev.

[68] Lederman SA, Paxton A (1998). Maternal reporting of prepregnancy weight and birth outcome: consistency and completeness compared with the clinical record. Matern Child Health J.

[69] Matsuda J, Yokota I, Iida M, Murakami T, Naito E, Ito M (1997). Serum leptin concentration in cord blood: relationship to birth weight and gender. J Clin Endocrinol Metab.

